# Retinoic acid reduces migration of human breast cancer cells: role of retinoic acid receptor beta

**DOI:** 10.1111/jcmm.12256

**Published:** 2014-04-10

**Authors:** Marina Inés Flamini, Gisel Valeria Gauna, Mayra Lis Sottile, Beatriz Silvina Nadin, Angel Matias Sanchez, Laura María Vargas-Roig

**Affiliations:** a Tumor Biology Laboratory, Institute of Medicine and Experimental Biology of Cuyo, National Research Council of ArgentinaMendoza, Argentina; b School of Medical Sciences, National University of CuyoMendoza, Argentina; c Reproduction and Lactation Laboratory, Institute of Medicine and Experimental Biology of Cuyo, National Research Council of ArgentinaMendoza, Argentina

**Keywords:** retinoic acid, RARβ, moesin, FAK, cell migration, breast cancer cells

## Abstract

Breast cancer is the most common malignancy in women and the appearance of distant metastases produces the death in 98% of cases. The retinoic acid receptor β (RARβ) is not expressed in 50% of invasive breast carcinoma compared with normal tissue and it has been associated with lymph node metastasis. Our hypothesis is that RARβ protein participates in the metastatic process. T47D and MCF7 breast cancer cell lines were used to perform viability assay, immunobloting, migration assays, RNA interference and immunofluorescence. Administration of retinoic acid (RA) in breast cancer cells induced RARβ gene expression that was greatest after 72 hrs with a concentration 1 μM. High concentrations of RA increased the expression of RARβ causing an inhibition of the 60% in cell migration and significantly decreased the expression of migration-related proteins [moesin, c-Src and focal adhesion kinase (FAK)]. The treatment with RARα and RARγ agonists did not affect the cell migration. On the contrary, the addition of the selective retinoid RARβ-agonist (BMS453) significantly reduced cell migration comparable to RA inhibition. When RARβ gene silencing was performed, the RA failed to significantly inhibit migration and resulted ineffective to reduce moesin, c-Src and FAK expressions. RARβ is necessary to inhibit migration induced by RA in breast cancer cells modulating the expression of proteins involved in cell migration.

## Introduction

Breast cancer is the most common malignancy in women, with ∽1.38 million new patients and 459,000 deaths/year worldwide (http://globocan.iarc.fr/). Despite improvements in early diagnosis and surgical and adjuvant systemic therapies for breast cancer, the mortality rate has remained high. It is therefore necessary to continue searching for novel approaches to breast cancer prevention, early detection and treatment.

Although at early stages breast cancer could be a well-curable disease, when metastasis occurs, the prognosis is severe. Metastasis constitutes the final step of the neoplasic progression and it became in the primary cause of death from solid tumours [1]. Therefore, the detailed knowledge of the molecular actions linked to the metastasis process is critical for the development of novel therapeutic strategies in oncology.

Retinoids are a family of signalling molecules chemically related to vitamin A (retinol). At present, little is known about the possible effects of retinoids, specifically, the retinoic acid (RA) role in breast cancer progression and metastasis. Retinoic acid, an active metabolite of vitamin A, plays essential roles in development, differentiation, cell growth and cellular homoeostasis [2,3]. All-*trans*, 9-*cis*, and 13-*cis*-RA are three stereoisomers of the RA [4]. They exert their metabolic effects mainly through the nuclear receptors: retinoic acid receptors (RAR) and the retinoic X receptors, both belong to the nuclear receptors superfamily [5].

The RARβ has become a particularly interesting target in cancer research. During cancer progression, *RAR*β gene deletion or promoter hypermethylation frequently occurs. It has been suggested that RARβ re-expression can restore RA-mediated growth control, indicating that the anticancer action of retinoids is mediated by RARβ [6–8]. Consequently, RARβ has been proposed as a tumour suppressor. However, the mechanism underlying its antitumour action has not yet revealed.

The *RAR*β promoter contains a high affinity RA-responsive element RARE [5,9,10], which is associated with the transcriptional activation of RARβ by RA in a variety of cells [5]. It has been observed an important reduction in *RAR*β mRNA expression in different types of human carcinomas including breast carcinoma [11–13].

Loss of *RAR*β gene expression has been reported in ∽50% of invasive breast carcinomas and it has been proposed as an essential player in the conversion of non-invasive breast cancer into invasive disease [13–16]. The *RAR*β tumour suppressor gene is the only RAR whose levels decrease drastically in many tumour types, but when RARβ get re-expressed the clinical response might be improved [17].

Genetic and epigenetic alterations may originate a progressive decrease in *RAR*β mRNA expression and the lack of RA response during breast carcinogenesis [12,13]. Recent studies have found that lack of RARβ is more often because of DNA methylation affecting the RARβ promoter [6,8,18]. The high frequency of hypermethylation in the *RAR*β promoter also suggests that epigenetical changes may confer a survival advantage to the disseminated cells at the distant site [19].

Recently, our laboratory, in accordance with other authors, has shown that *RAR*β methylation in primary breast tumours correlated with lymph node invasion and metastasis [20]. The metastatic process requires the acquisition of invasive properties such as motility, invasion and remodelling of the extracellular matrix (ECM). A key regulator of ECM signals is the actin-binding protein moesin, which belongs to the ezrin/radixin/moesin (ERM) family. Activated moesin triggers the de-polymerization of actin fibres and the re-assembly of microfilaments towards the cell membrane edge, leading to the formation of cortical actin complexes and specialized cell membrane structures implicated in the generation of the cellular locomotive force [21]. Interestingly, ERM proteins have been recently reported as key regulators of metastasis in aggressive cancers [22,23]. Another ECM controller is the focal adhesion kinase (FAK), a non-receptor protein tyrosine kinase involved in cell attachment, migration, invasion, crucial steps for cancer development and metastasis [24,25]. Focal adhesion kinase overexpression has been described in human cancers and is related to invasive potential of tumour cells and poor prognosis [24,26,27]. It has been shown that FAK suppression is associated with decreased mobility and metastastic capacity in breast cancer cells [28]. Focal adhesion kinase is activated by c-Src, a non-receptor tyrosine kinase that recruited FAK to the c-Src complex [29]. In addition, the activation of c-Src has been significantly associated with tumour progression and aggressive features [30,31]. Activated FAK–Src complex mediates the phosphorylation of multiple adhesion components involved in the dynamic regulation of cell motility.

The purpose of our study was to investigate the effects of RA on human breast cancer cells migration, underlying molecular mechanisms involved and the possible roles of RARβ. To this aim, we studied the consequences of RA treatment on the expression of moesin, c-Src and FAK and actin remodelling in breast cancer cells.

## Materials and methods

### Cell cultures and treatments

The human breast carcinoma cell lines T47D and MCF7 were obtained from the American Type Culture Collection (Rockville, MD, USA). T47D and MCF7 cells were routinely grown in RPMI 1640 supplemented with l-glutamine (2 mM) and 10% foetal bovine serum. All-*trans*-RA was obtained from Sigma Chemical Co. (St. Louis, MO, USA). Retinoic acid stock solution was dissolved in dimethyl sulfoxide (DMSO) at a concentration of 10^−2^ M and maintained at −20°C, protected from light and in an inert atmosphere. The synthetic agonist retinoids, selective for RARα (BMS753), RARβ (BMS453) and RARγ (BMS961), and synthetic antagonist retinoids selective for RARα (BMS195614; Tocris Bioscience, Bristol, UK) were kindly provided by Dr. Hinrich Gronemeyer (IGBMC, Illkirch, France). Agonist and antagonist retinoids were diluted in ethanol and added to the culture medium to give a final concentration of 10^−6^ M. In control cultures, the DMSO or ethanol vehicle was added at the same final dilution. All experiments with retinoids were performed in reduced room light.

### Viability assay

The MTT [3-(4,5-dimethylthiazol-2-yl)-2,5-difeniltetrazol] (Sigma Aldrich, St. Louis, MO, USA) was dissolved at a concentration of 5 mg/ml in RPMI culture medium. The working solution was 0.1 mg/ml MTT. Cell lines were seeded into 96 well plates at a density of 13,000 cells/well. 24 hrs later, the RA dose-dependency treatment (10^−7^/10^−5^ M) was performed. After 72 hrs, the medium was removed and the cells were incubated with 100 μl MTT/well (0.5 mg/ml) for 3–4 hrs. MTT was removed and the formazan crystals rings were dissolved in 100 μl of DMSO. Absorbance at 570 nm was measured by using a microplate reader (MULTISKAN EX; Thermo Scientific, Lafayette, CO, USA).

### Cell migration assays (wound healing assay)

Scratch wound assay was conducted to assess the influence of RA cell migration. T47D and MCF7 cells were seeded in six well plates and incubated until 70–80% confluence. Wounds were made in the monolayers by scratching the surface with a pipette tip (1000 μl) as uniformly and straight as possible. Then, the cells were washed and 2.0 ml of RPMI containing steroid-deprived fetal bovine serum were added. Cytosine β-d-arabinofuranoside hydrochloride (Sigma-Aldrich; 10 μM), a selective inhibitor of DNA synthesis which does not inhibit RNA synthesis, was used 1 hr before the test substance was added. Cell migration was monitored for 72 hrs. Every 12 hrs, fresh medium and treatment was replaced. Digital images from cells were taken and the distance of migration was then analysed by phase-contrast microscopy.

### Immunoblotting

Cell lysates were separated by SDS-PAGE. Antibodies used were: Mouse Anti-FAK (610088) and Mouse Anti-moesin (clone 38, 610401; BD Transduction Laboratories, Milano, Italy), Mouse Anti-c-Src (sc-5266) and Rabbit Anti-RARβ (C-19): sc-552 (Santa Cruz Biotechnology, Santa Cruz, CA, USA). Primary and secondary antibodies were incubated with the membranes by using standard techniques. Immunodetection was accomplished by using enhanced chemiluminescence. The images were captured by using ChemiDoc™ XRS+ System with Image Lab™ Software #170-8265 (Bio-Rad, Hercules, CA, USA).

### Transfection experiments

The synthetic small interfering RNA for RARβ: sc-29466 was from Santa Cruz Biotechnology and control siRNA (D-001810-01-05) was obtained from Dharmacon (Thermo Fisher Scientific Inc.). T47D and MCF7 cells (60–70% confluent) were transfected with 50–75 nM of target siRNA or control siRNA by using Lipofectamine (Invitrogen, Carlsbad, CA, USA). The cells were treated 24 hrs after siRNA transfection. Transfection efficiency was checked for expression of RARβ by imunoblotting and it was optimal at 48 hr.

### Cell adhesion assay

Five hundred thousand cells per well were seeded into 6-well plates on coverslips previously coated with 1% sterile gelatin (Sigma-Aldrich) and exposed to different treatments. The cells were incubated at 37°C (in tissue culture incubator) and after 1 hr, the plates were shaken for 1 min. at 150 r.p.m. and washed with PBS to remove any non-adherent cells. The attached cells were fixed with 4% formaldehyde and stained with Giemsa. Cells attached images were captured and counted by using a Nikon Eclipse E200 microscope coupled to a high-resolution 590CU 5.0M CCD digital camera.

### Immunofluorescence

T47D and MCF7 cells were grown on coverslips and exposed to treatments. Cells were fixed with 4% paraformaldehyde for 30 min. and permeabilized with 0.1% Triton for 5 min. Blocking step was performed with 3% bovine serum albumin solution for 30 min. at room temperature. Cells were incubated with the first antibody against moesin (clone 38) overnight at 4°C. After washing, the cells were incubated with goat antimouse IgG-Alexa Fluor 488 (A-11001; Invitrogen) for 1 hr at room temperature. The cells were washed and then stained with Texas Red-phalloidin (Sigma-Aldrich) to reveal actin and the nuclei counterstained with 4′-6-diamidino-2-phenylindole (DAPI; Sigma-Aldrich). The coverslips cells were mounted with Vectashield mounting media (Vector Laboratories, Burlingame, CA, USA). Immunofluorescence images were captured by using a Nikon Eclipse E200 microscope (Tokyo, Japan) coupled to a high-resolution 590CU 5.0M CCD digital camera.

### Evaluation of cellular morphology

Immunofluorescence images stained with Texas red-phalloidin (actin) were used to quantify the effects, of cells with a static and a migratory phenotype. Cells simultaneously showing loss of stress fibres, progressive localization of actin towards the edge of the cell membrane, and presence of numerous stress fibre arcs were considered as having a migratory phenotype [32]. Conversely, a static phenotype was characterized by actin fibres arranged longitudinally through the major axis of the cell shape [32].

### Statistical analysis

D'Agostino & Pearson omnibus normality test was used to determine whether the samples have normal distribution. Statistical analysis of the data was performed with one-way anova followed by Turkey-Kramer Multiple-Comparisons test or the non-parametric Kruskal–Wallis test (GraphPad PRISM program version 5.0, San Diego, CA, USA). *P* < 0.05 was considered as statistically significant. All values were expressed as mean ± SD.

## Results

### Effects of RA on breast cancer cells viability

**Figure 1:**
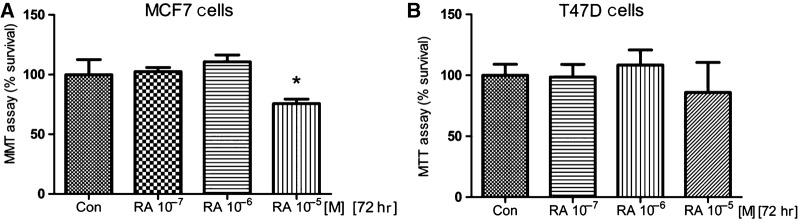
(A and B) The cellular lines MCF7 and T47D were seeded at 13,000 cells per well and treated with RA (10^−7^/10^−5^ M) for 3 days. The results were expressed as percentage (%) of survival cells. All data shown were representative of three independent experiments. Error bars indicate standard deviations. *P < 0.05 *versus* Con.

Retinoids are known to inhibit the growth of breast cancer cells [2,11]. However, the MTT assay showed that 72 hrs of RA treatment at increasing concentrations (10^−7^–10^−5^ M) did not affect T47D and MCF7 cells viability, but at the highest concentrations (10^−5^ M) in MCF7 cells, the viability decreased 20% compared with untreated (control) cells (Fig.1A and B).

### RA reduces MCF7 and T47D cells migration

**Figure 2:**
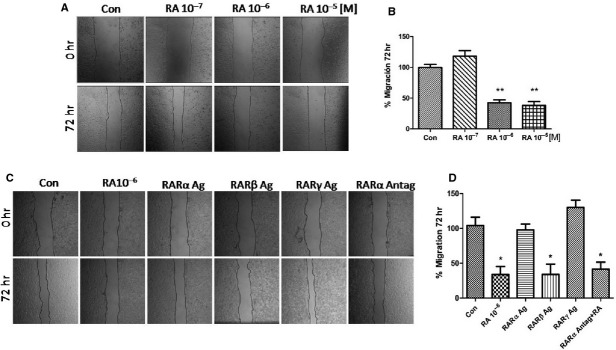
(A) MCF7 cells were treated with retinoic acid (RA) in different concentrations (10^−7^/10^−5^ M) and cell migration was imaged after 72 hrs. (B) Gap closure was quantified with the use of NIH image J software. *P < 0.05 *versus* Con. (C) T47D cells were treated with RA (10^−6^ M) and the synthetic agonist retinoids, selective for RARα Agonist (BMS753), RARβ Agonist (BMS453) and RARγ Agonist (BMS961), and the synthetic antagonist retinoids selective for RARα (BMS195614) plus RA (10^−6^ M). All retinoids were incubated at 10^−6^ M for 72 hrs. Cell migration was imaged after 72 hrs. (D) Gap closure was quantified with the use of NIH image J software. *P < 0.05 *versus* Con. These experiments were performed in triplicates and representative images are shown.

The effect of RA on breast cancer cell migration was then tested in a dose–response experiment. To distinguish cell migration from cell proliferation, Cytosine-β-d-arabinofuranoside hydrochloride (10 μM), a selective inhibitor of DNA strand separation that does not block RNA synthesis, was used to arrest cell proliferation. After partially scraping out MCF7 cells from the cell culture dish, we monitored the movement of the remaining cells for the following 72 hrs. After 72 hrs, 10^−6^ and 10^−5^ M of RA significantly inhibited the migration of MCF7 cells towards the scraped area ‘the wound healing’ compared with control untreated cells (Fig.2A and B). It is important to note that the 60% of cell migration inhibition started from RA 10^−6^ M, but at the same concentration the cell viability was not affected (Figs1A and 2A, B). Similar results were obtained in T47D cellular line (data not shown).

### The synthetic retinoid RARβ agonist, BMS 453, inhibits breast cancer cells migration

To determine which subtype of RAR is involved in RA-induced migration inhibition, we tested the effects of selective synthetic retinoid agonists, for RARα (BMS753), RARβ (BMS453) and RARγ (BMS961), and the RARα-selective antagonist (BMS195614).

Treatment with RA 10^−6^ M for 72 hrs significantly reduced T47D breast cancer cells migration (Fig.2C and D). Retinoic acid receptor α-selective antagonist (BMS195614) in combination with RA did not affect the cell movement, suggesting that RARα receptor is not required for RA effects on cell migration. The RARβ-selective agonist (BMS453), but not RARα- or RARγ-selective agonists (BMS753 and BMS961, respectively), significantly reduced the cell migration to levels comparable to inhibition by RA, indicating that RARβ is involved in RA-inhibited cell migration (Fig.2C and D). Similar results were obtained in MCF7 cellular line (data not shown).

### RARβ protein expression is regulated by AR in breast cancer cells lines

**Figure 3:**
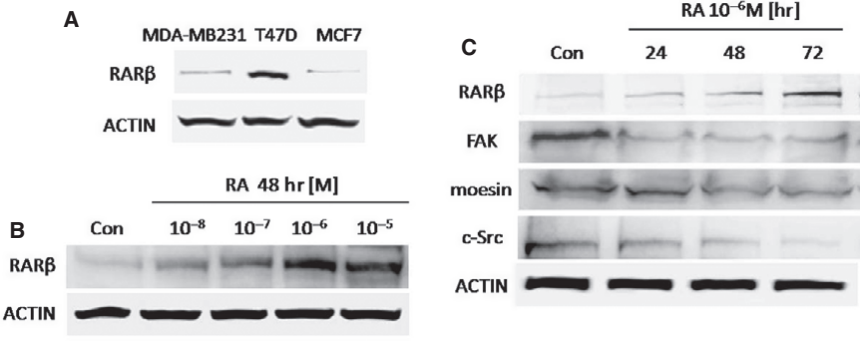
(A) Western blot analysis for RARβ protein was performed in 30 μg of total lysate of untreated MDA-MB231, T47D and MCF7 breast cancer cells. (B) Western blots show total cell amount of RARβ in MCF7 treated with different concentrations of retinoic acid (RA; 10^−8^/10^−5^ M) for 48 hrs. (C) MCF7 breast cancer cells were treated with RA (10^−6^ M) for 24, 48, 72 hrs and the RARβ, FAK, moesin, and c-Src expression are shown. Actin expression is shown in the lower boxes as loading control. The experiments were repeated three times with consistent results and representative images are shown. Densitometric quantifications of all the blots (including those not shown) were performed and the relative mean ± SD of each condition are presented in graph as supplemental data online Fig. S1A–C.

The expression of RARβ protein varies among breast cancer cell lines. Zhang *et al*. did not detect RARβ expression in five breast cancer cell lines and showed that the treatment with RA could induce its expression [33]. We have evaluated the RARβ protein expression in three breast cancer cells lines under basal conditions (MDA-MB231, T47D and MCF7). We verified that RARβ protein expression was higher in T47D cells compared with MDA-MB231 and MCF7 cells (Fig.3A). This result is in accordance with other authors who have shown that *RAR*β gene expression may be lost by hypermethylation in breast cancer cells lines such as MDA-MB231 or MCF7 cells [6].

Next, we investigated the expression of RARβ in MCF7 cells after a RA dose-dependent treatment. It has been reported that RARβ is weakly expressed in MCF7 cells but it can be induced by RA treatment [6]. Our results demonstrate that RA administration on MCF7 cells can induce RARβ protein expression with a peak at 10^−6^ M after 72 hrs (Fig.3B and C), which is consistent with the kinetics of RA-induced migration inhibition.

### RA reduces the expression of moesin, c-Src and FAK, which correlates with its migration-inhibitory effect

Based on our previous findings in which the actin-regulatory protein moesin and the kinases c-Src and FAK resulted to be essential proteins for the migration induced by different hormones [34–36], we have investigated if these proteins could be involved in the biological activities of RA resulting in reduced breast cancer cells migration. Time-dependent treatment (24–72 hr) with RA 10^−6^ M significantly reduced the expression of moesin, c-Src and FAK (Fig.3C). Interestingly, the expression levels of moesin, FAK and c-Src in these cells were inversely correlated with those of RARβ (Fig.3C).

### RA fails to impaired cellular migration when RARβ is silenced

**Figure 4:**
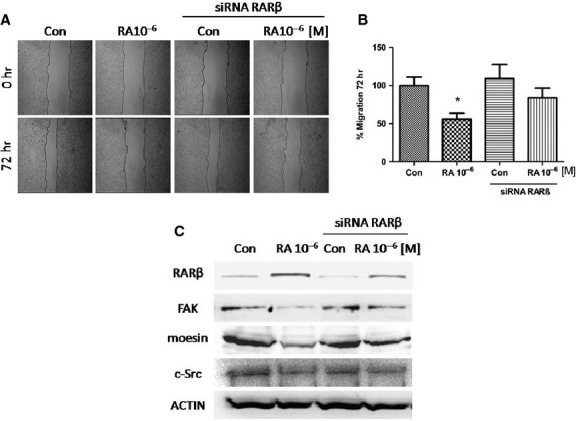
MCF7 cells were exposed to retinoic acid (RA; 10^−6^ M/72 hr) and another group was first transfected with a specific RARβ siRNA and then treated or not with RA (10^−6^ M/72 hr). (A) Cell migration was imaged after 72 hrs. (B) Gap closure was quantified with the use of NIH image J software. *P < 0.05 *versus* Con. (C) Western blot analysis for RARβ, FAK, moesin and c-Src. Actin expression is shown in the lower boxes as loading control. These experiments were performed in triplicates and representative images are shown. Densitometric quantifications of all the blots (including those not shown) were performed and the relative mean ± SD of each condition are presented in graph as supplemental data online Fig. S2.

We next treated MCF7 cells with RA (10^−6^ M/72 hr) and another group was first transfected with a specific RARβ siRNA and then exposed to or not with RA (10^−6^ M/72 hr). After performing a cell migration assay, we found that RA was able to significantly decrease cell migration, but failed to inhibit migration in cells transfected with RARβ siRNA, indicating that the inhibition of cell migration by RA is mediated by the RARβ pathway (Fig.4A and B).

To demonstrate if RARβ mediates RA effects on cell movement, we have studied the expression of moesin, c-Src and FAK proteins in MCF7 cells treated with RA after RARβ silencing. Therefore, RARβ silencing impaired the reduction in these proteins by RA indicating a RARβ-dependent behaviour. In other words, RA reduced the expression of these proteins through RARβ (Fig.4C).

### RA reduces migration and adhesion in T47D cells

**Figure 5:**
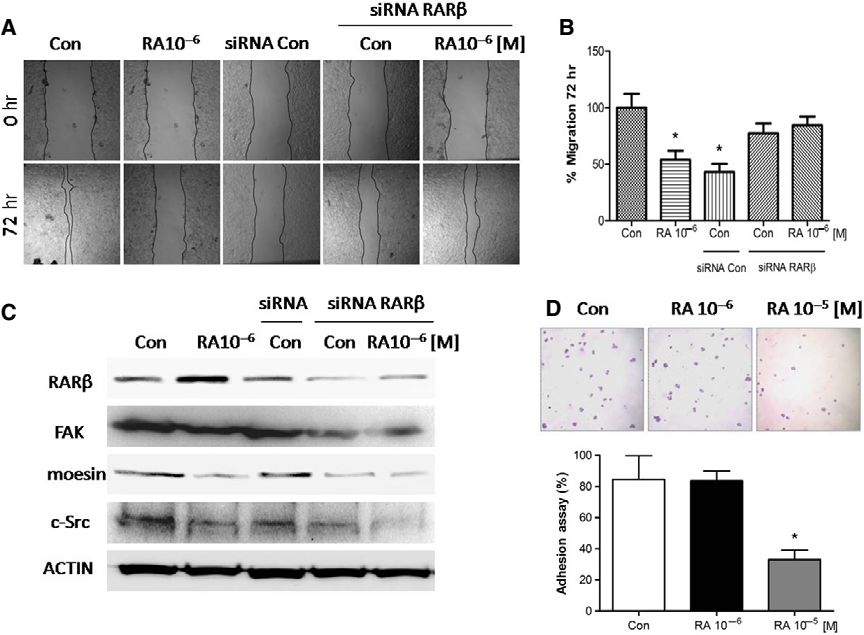
T47D cells were exposed to retinoic acid (RA; 10^−6^ M/72 hr) and another group was first transfected with a control siRNA or specific RARβ siRNA and then treated or not with RA (10^−6^ M/72 hr). (A) Cell migration was imaged after 72 hrs. (B) Gap closure was quantified with the use of NIH image J software. *P < 0.05 *versus* Con. (C) Western blot analysis for RARβ, FAK, moesin, c-Src and Actin as loading control was performed. (D) Representative images of T47D cell adhesion to gelatin after RA treatment (10^−6^/10^−5^ M) for 72 hrs are shown. These experiments were performed in triplicates and representative images are shown. Densitometric quantifications of all the blots (including those not shown) were performed and the relative mean ± SD of each condition are presented in graph as supplemental data online Fig. S3.

The treatment with RA (10^−6^ M/72 hr) caused 50% inhibition of cell migration in T47D cells (Fig.5A and B). Retinoic acid failed to inhibit the cell migration after RARβ silencing, suggesting that RA could suppress the cell migration through RARβ (Fig.5A and B). Furthermore, untreated cells transfected with a control siRNA showed a lower cell migration than RARβ-silenced cells (Fig.5A and B).

On the contrary to the expected, RARβ-siRNA transfected T47D cells showed a similar decrease in moesin, FAK and c-Src expressions after treatment with RA (Fig.5C).

In addition to reduce T47D cells migration, the treatment with RA (10^−5^ M/72 hr) significantly inhibited T47D cell adhesion to gelatin (Fig.5D).

### RA induces a static phenotype of migration through inhibition of Moesin

**Figure 6:**
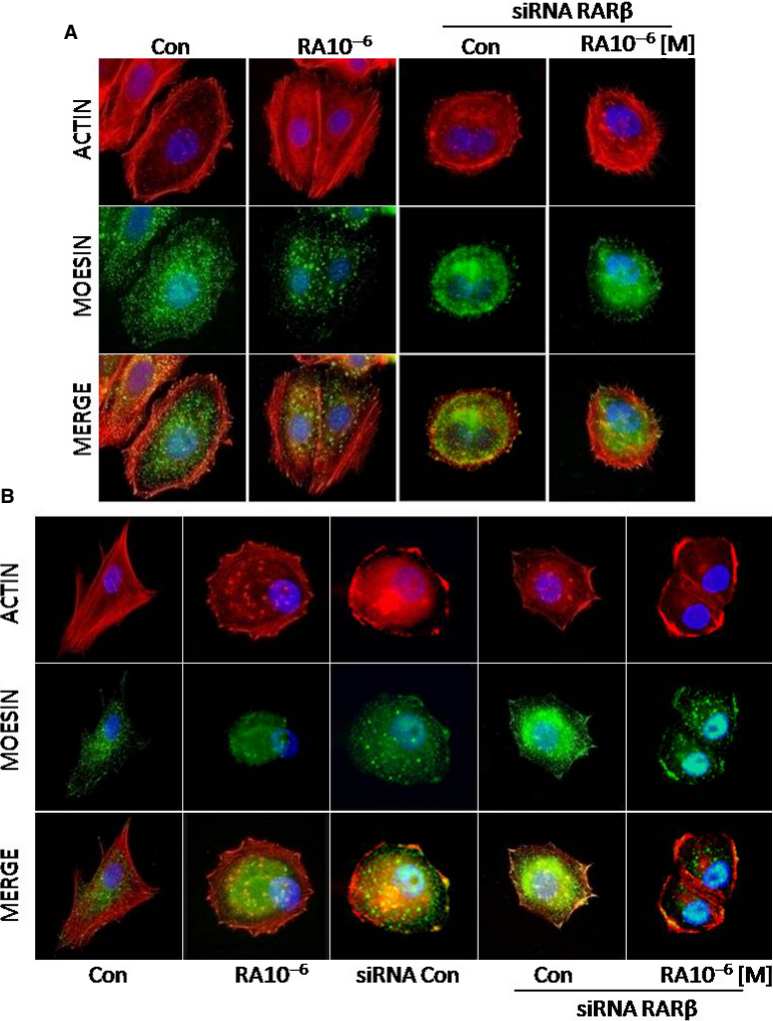
(A and B) MCF7 and T47D cells were exposed to retinoic acid (RA; 10^−6^ M/72 hr) and another group was first transfected with a specific RARβ siRNA and then treated or not with RA (10^−6^ M/72 hr). Then the cells were stained with anti-moesin (clone 38) linked to Alexa Fluor 488 (green), actin was stained with phalloidin linked to Texas Red (red) and nuclei were counterstained with DAPI (blue). All the experiments were repeated three times with consistent results, and the representative images are shown. The microphotographs were taken with a 100 × objective.

To better understand the dynamics of breast cancer cells migration and the different behaviour observed between untreated and RA treated cells, we next investigated changes in the cytoskeleton organization induced by RA treatments. MCF7 and T47D cells non transfected/transfected with siRNA RARβ were exposed to RA (10^−6^ M/72 hr). We have examined through inmunofluorescence experiment the expression and localization of the modulator protein moesin with Alexa fluor 488 (green) and actin fibres with the specific marker Texas red-phalloidin (red). In control cells (without treatment), actin filaments arranged longitudinally along the major axis (Fig.6A and B) and moesin showed a diffuse distribution throughout the cytoplasm (Fig.6A and B). The treatment with RA significantly reduced the expression of moesin, which changed its spatial organization, showing an increment of stress fibres and a progressive localization of the protein towards the nucleus. At this localization is difficult for moesin to form cortical actin complexes, moesin aggregates or specialized cell membrane structures, which are present in the cell membrane to induce cell motility (Fig.6A and B).

When the cells were transfected with the specific RARβ siRNA, RA failed to reduce moesin expression and was not effective to induce moesin re-distribution (Fig.6A and B).

**Figure 7:**
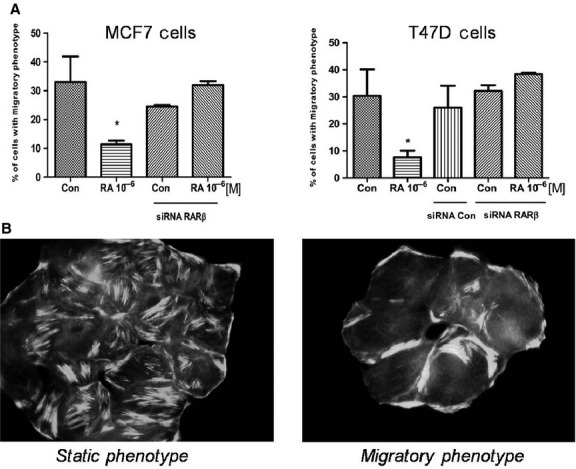
(A) Immunofluorescence images of MCF7 and T47D cells stained with Texas Red-phalloidin (actin) were used to quantify the effects, of cells with a static and a migratory phenotype. The results were expressed as percentage (%) of Cells with Migratory Phenotype. (B) Representative images of Static and Migratory Phenotype of T47D cells are shown. All the experiments were repeated three times with consistent results, and the representative images are shown. The microphotographs were taken with a 100 × objective.

Furthermore, after 72 hrs of RA 10^−6^ M incubation most of MCF7 and T47D cells showed a static phenotype (Fig.7A). The static phenotype is generally characterized by numerous actin fibres longitudinally arranged through the major axis of the cell shape. In contrast, the migratory phenotype exhibits loss of stress fibres, progressive localization of F-actin towards the edge of the cell membrane, and numerous stress fibres arcs (Fig.7A). Thus, after 72 hrs of RA incubation, a significant difference in the number of cells with a migratory phenotype was observed between controls and RA treated cells. Moreover, this inhibitory effect was extremely limited in the RARβ down-regulated group (Fig.7A), determining that RARβ mediates the effect of RA on moesin down regulation and cellular localization.

## Discussion

Retinoids have long been investigated in preclinical models, and clinical data have already supported the potential of these compounds in cancer prevention and treatment [37–39].

Retinoic acid is being increasingly included in both chemopreventive and therapeutic schemes for various tumour diseases including breast cancer [39–41]. In general, RA is believed to inhibit carcinogenesis by blocking the promotion of initiated or transformed cells by three mechanisms: induction of apoptosis, arrest of tumour growth and/or differentiation of cancer cells [41]. Growth inhibition of breast cancer cells by RA has been associated with the induction of RARβ, which may act as a tumour suppressor and appears to be down-regulated in breast cancer cells and, conversely, up-regulated in normal mammary epithelial cells [33].

Several studies have also reported that RA may inhibit invasion and metastasis in diverse types of cancer such as breast [42] and colon cancer cells [43]. However, the mechanism by which RA blocks the late stages of carcinogenesis is largely unknown. In addition, limitations in the successful of prevention and treatment of solid tumours with retinoids may be related to the epigenetic silencing of RARβ [4].

The multifaceted approach of this study was an attempt to provide a comprehensive analysis of the RA effects on the migration capability by using two human breast cancer cell lines. We have demonstrated the ability of RA to inhibit breast cancer cell migration in a dose- and time-dependent manner; others studies using various cell lines also demonstrated a similar effect [44–47]. In agreement with other authors, we have demonstrated the cellular motility inhibition by RA in breast cancer cells in absence of growth inhibition [48]. The possible mechanisms implicated in the RA anti-migratory effect were further investigated, and our data showed that RA treatment up-regulates RARβ expression and down-regulates moesin, FAK, and c-Src (proteins related to migration) expression in MCF7 cells (which have very low RARβ expression in basal conditions by promoter methylation).

Numerous studies indicate that FAK could be an important player in signalling cascades associated with cancer progression and metastasis [49,50]. Increased FAK expression has been correlated with increased cell motility, invasiveness and proliferation [51,52]. Moesin has also been proposed as key regulator of metastasis in aggressive cancers [22,23]. Therefore, we hypothesize that RA reduces moesin, FAK and c-Src expressions in breast carcinoma MCF7 cell lines by RARβ. In fact, RARβ expression was induced by treatment with RA and RARβ silencing abolished the ability of RA to reduce the expression of moesin, FAK and c-Src in these cells. According to previous reports, the anti-cancer potential of RA is mainly mediated by RARβ [7,53] which increases the expression of the migration-related protein E-cadherin [46].

In T47D cells, as in MCF7 cells, we observed that RA reduced significantly cell migration (55%) but this reduction only represented 20–30%, when RARβ was silenced. Contrary to our expectations, after RARβ silencing, RA reduced moesin, c-Src and FAK expressions but cells express a migratory phenotype in which moesin is localized near the edge of the cell membrane forming specific cellular structures for migration (cortical actin complexes, pseudopodia, and membrane ruffles). Therefore, in T47D cells, the effect of RA on cell migration may be mediated not only by RARβ but also by RARα and/or RARγ. On the other hand, we have kept in mind that moesin, c-Src and FAK are three of the several proteins related to motility and that RARβ could be involved in the modulation of one or more of these proteins (talin, vinculin, paxilin and/or contactin).

In cancer cells with a down-regulated RARβ expression, RA resulted ineffective to reduce cellular migration, suggesting that tumour cells could silence RARβ to facilitate the escape of the tumour triggering the metastatic process. This is the first time that silencing of RARβ was associated with cell migration, which may contributes to its antitumour activity. In agreement with other research groups, we consider that RARβ promoter hypermethylation may confer a survival advantage to the disseminated cells at the distant site [19].

Some authors have demonstrated that RA and other biologically active retinoids inhibit cellular migration in several cell lines such as human colon carcinoma cells [46], and human breast cancer MCF7 and MDA-MB-231 cells [54,55]. Dutta *et al*. demonstrated that RA treatment caused a significant decrease in FAK expression in MCF7 cells and they suggest that it may contribute to the cell motility decreased [54]. Furthermore, in MDA-MB-231 cells, the same authors demonstrate that RA enters into the nucleus and regulates various signalling pathways including Integrin, FAK, ERK, PI-3K, NFkB and down-regulates pro-MMP-9 activity as well as its expression. They showed that the migration of MDA-MB-231 cells on fibronectin medium is retarded in presence of RA [55]. However, these authors did not explore which RA receptor subtype mediates the effects on cell migration and motility.

Alterations in the adhesion and motility properties of neoplastic cells may play a pivotal role in the development and progression of the malignant phenotype in various tumour types. Invasion is a multiphase process constituted by different coordinated interdependent steps, controlled by cross-talk mechanisms between cells and the extracellular microenvironment [56].

We verified by indirect immunofluorescence staining, that RA reduced the appearance of peculiar structures such as ruffles and pseudopodia and it affected the cytoskeletal rearrangement through reorganization of the stress fibres inhibiting the formation stress fibres arcs and lamellipodia that frequently occur in moving cells. Retinoic acid induced a static phenotype in MCF7 and T47D cells, with actin fibres longitudinally arranged through the major axis of the cell surface. Thus, after 72 hrs of RA incubation, a statistically significant reduction in the number of cells with a migratory phenotype was observed. Moreover, this inhibitory effect was extremely limited in RARβ down-regulated group. Our results indicate that RA may play a significant role in breast cancer development and progression modulating cellular migration and cytoskeletal reorganization *via* RARβ.

Chang *et al*. demonstrated that RA differentially regulates *de novo* synthesis of various ECM proteins in cultured human retinal pigment epithelial cells (RPE) [57]. The underlying mechanism may be, at least in part, becuase of the suppressive effects of RA on integrin b3 expression. In conclusion, RA appears to have the ability to alter the expression of ECM proteins, remodelling the cytoskeleton [57,58]. Therefore, we speculate that RA could modify the breast cancer cells phenotype from invasive to non-invasive. Du. *et al*. demonstrated that after RA treatment, twenty genes which were correlated with migration of RPE cells were down-regulated, whereas another four genes had been up-regulated [58]. They found that the expression of integrin genes were down-regulated by RA. Integrins are proteins highly related to FAK. The up-regulation of E-cadherin by RA prevents RPE cell migration through maintaining cell and tissue cohesion. Therefore, genes whose expressions are involved in motility may play an important role in migration and invasion [58].

The strong antimetastatic effect of RA suggests the importance to design treatments combining retinoids and other agents. Several retinoids are currently in clinical trials to treat or prevent breast cancer progression. Retinoids are effective inhibitors of breast cancer cells at the early stages of tumour progression, but their effectiveness diminishes when the tumours become more aggressive [59]. Therefore, to develop rational retinoid-based therapy for breast cancer, it becomes necessary to elucidate the molecular pathways activated by retinoids. Retinoic acid receptor β expression deficiency and inadequate responsiveness of retinoids *via* RARβ may reduce the treatment efficacy in patients with advanced breast tumours [59]. It is also becoming increasingly obvious that RARβ expression is lost early in carcinogenesis and it is epigenetically silenced [6] in many solid tumours, providing an opportunity for novel treatment strategies to be investigated by using retinoids together with epigenetic modifiers that promote re-expression of silenced genes. The limited treatment success with retinoids observed to date in the prevention and treatment of solid tumours may be related to the frequent epigenetic silencing of RARβ. Robust evaluation of RARβ and downstream genes may optimize the use of retinoids in solid tumours.

## Conclusion

Our study demonstrates that high concentrations of RA induce RARβ expression which mediates cell migration and cell motility inhibition in breast cancer cells. The results show the participation of RA in some properties of cancer cells involved in the metastatic process, like motility and adhesivity. More pharmacokinetic studies of retinoids are needed to elucidate other biological effects of retinoids in human tumour cells to design novel clinical trials for cancer therapy or strategies to prevent the malignant transformation.

## Supporting Information

**Figure S1** Densitometric quantification of western blot analysis for (**A**) RARβ protein of untreated MDA-MB231, T47D and MCF7 breast cancer cells. (**B**) RARβ in MCF7 cells treated with different concentrations of RA (10^−8^/10^−5^ M) for 48 hr. (**C**) RARβ, FAK, moesin, and c-Src expression in MCF7 cells treated with RA (10^−6^ M) for 24, 48, 72 hr. and the are shown.

**Figure S2** Densitometric quantification of western blot analysis for RARβ FAK, moesin and c-Src in MCF7 cells exposed to RA (10^−6^ M/72 hr) or transfected with a specific RARβ siRNA and then treated or not with RA (10^−6^ M/72 hr).

**Figure S3** Densitometric quantification of western blot for RARβ, FAK, moesin, c-Src in T47D cells exposed to RA (10^−6^ M/72 hr) or transfected with a control siRNA or specific RARβ siRNA and then treated or not with RA (10^−6^ M/72 hr)
