# Supplementary figures and images for: Retinoic acid reduces migration of human breast cancer cells: role of retinoic acid receptor beta
Source: J Cell Mol Med. 2014 Apr 10;18(6):1113–23. doi: 10.1111/jcmm.12256 (PMC4508151; doi:10.1111/jcmm.12256)

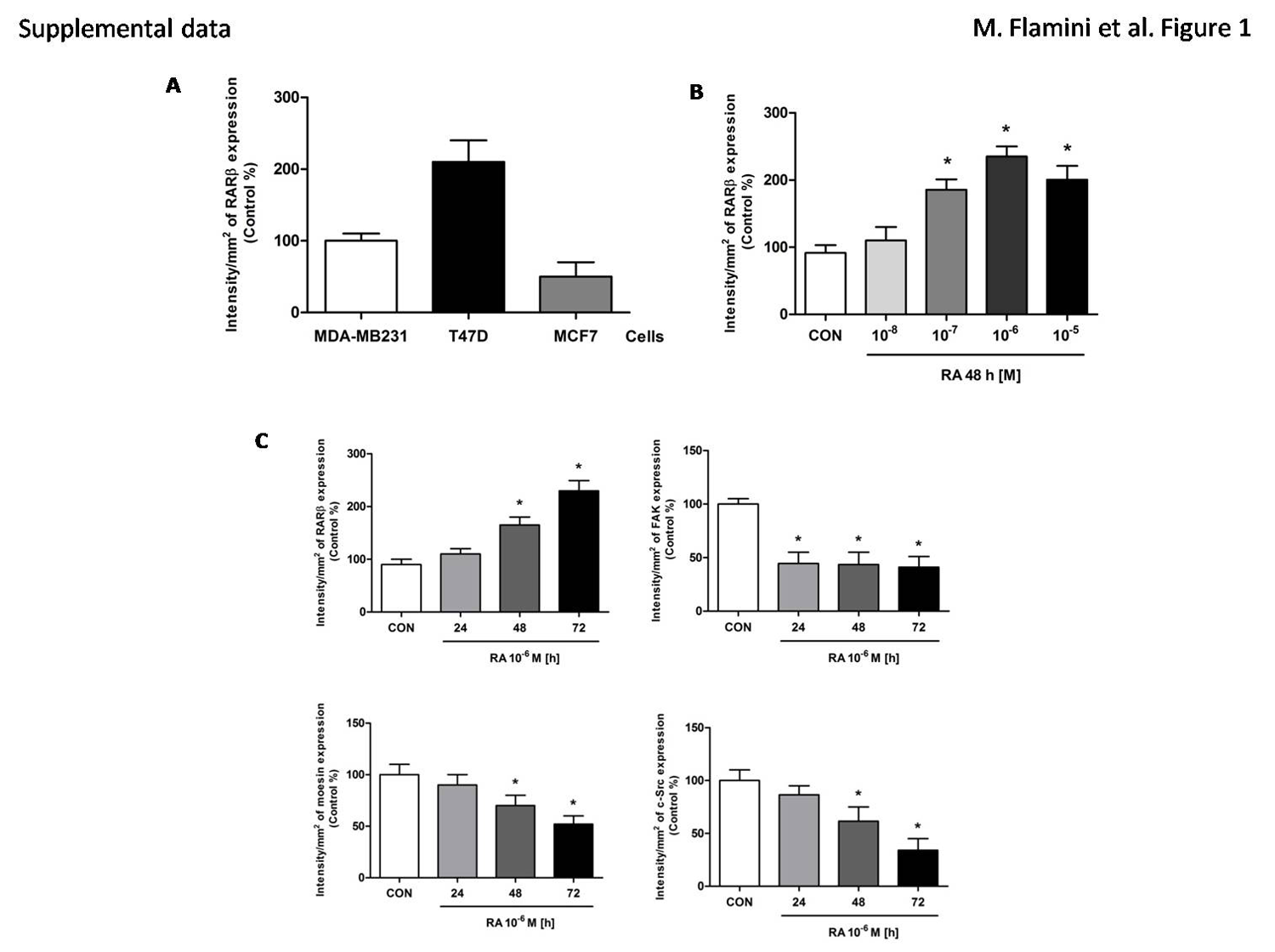

Supplement: Supplementary file 1 [file jcmm0018-1113-sd1.tif]

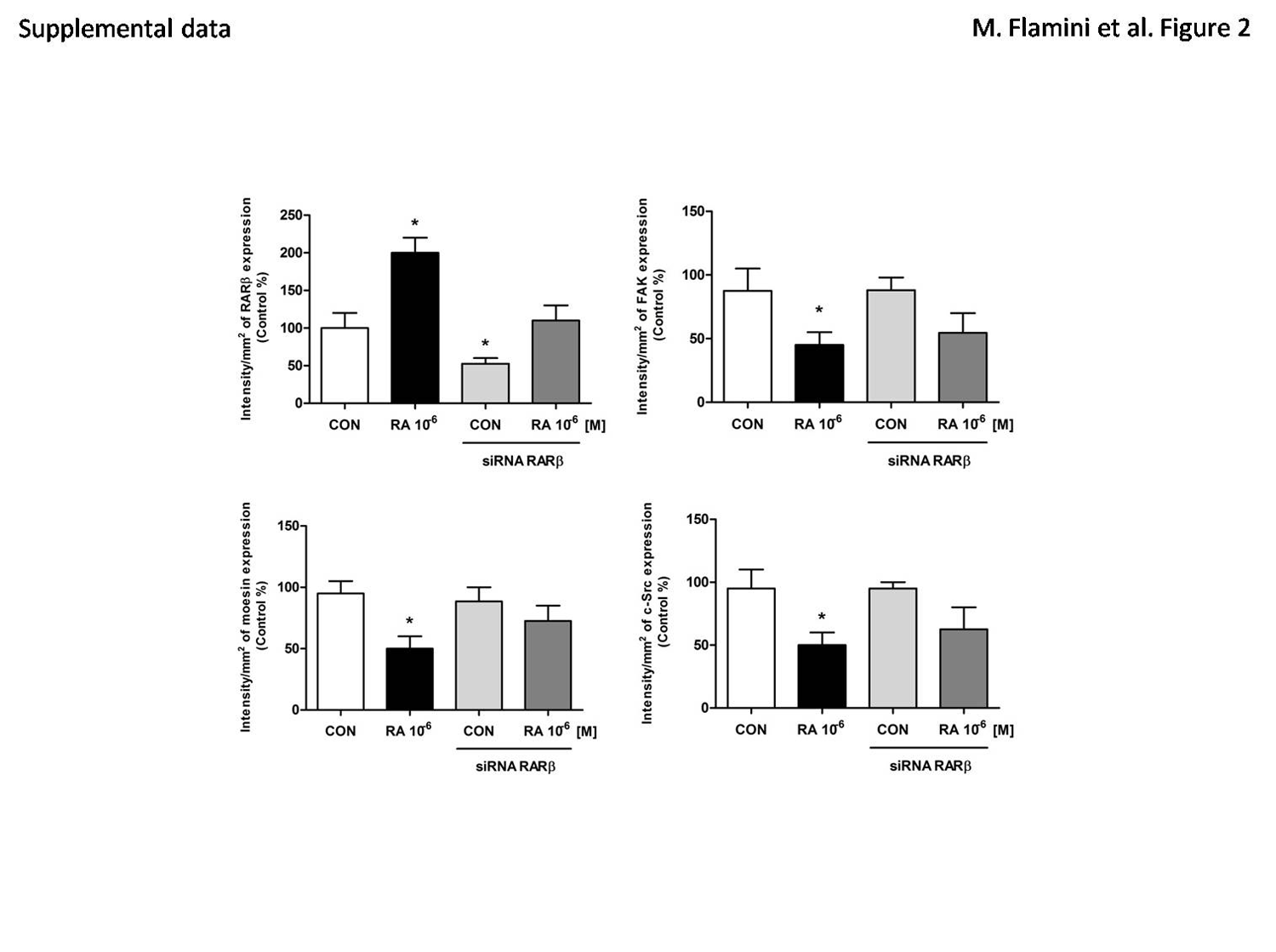

Supplement: Supplementary file 2 [file jcmm0018-1113-sd2.tif]

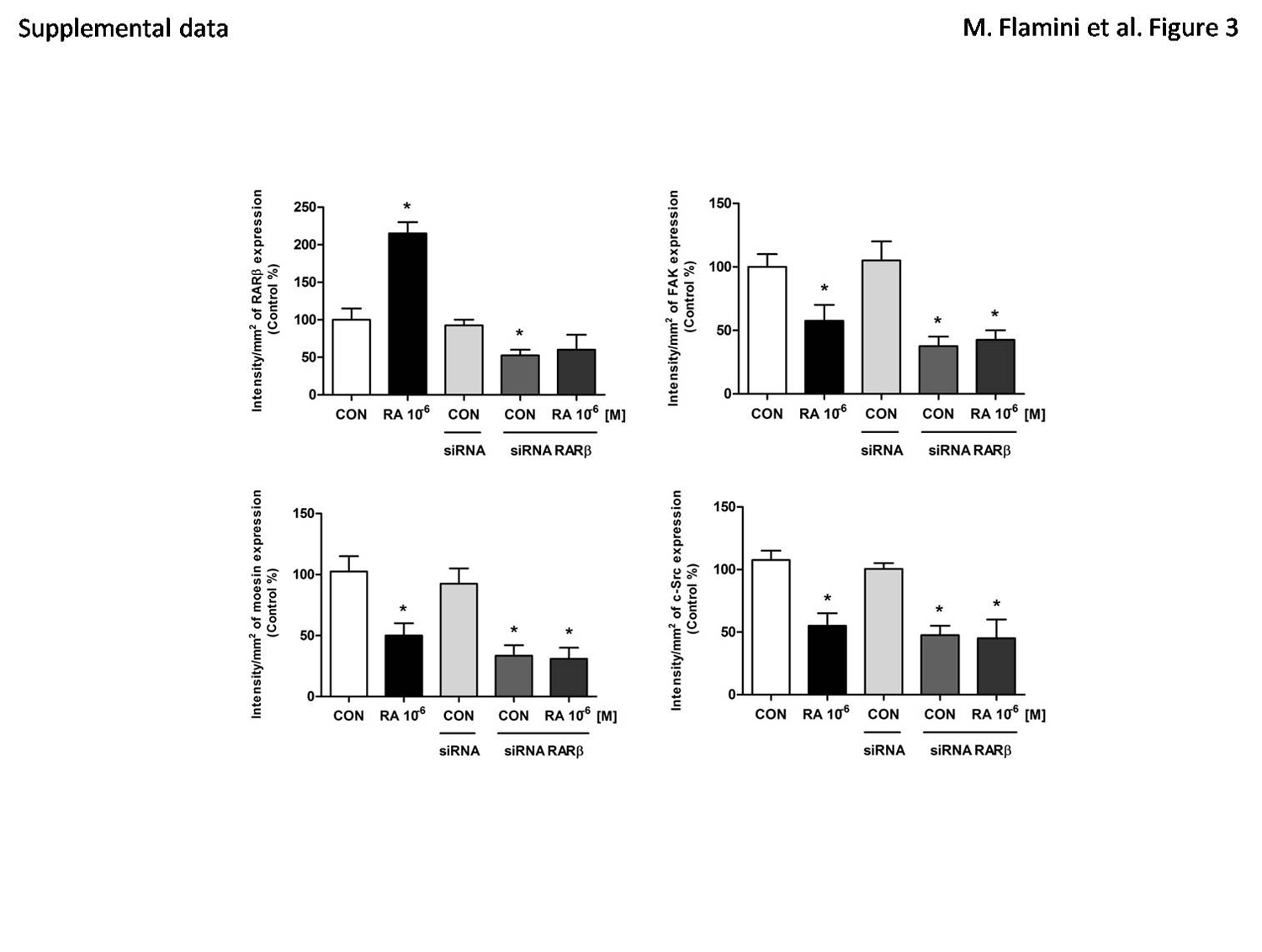

Supplement: Supplementary file 3 [file jcmm0018-1113-sd3.tif]
